# Reversal of sorafenib resistance in hepatocellular carcinoma by curcumol: insights from network pharmacology, molecular docking, and experimental validation

**DOI:** 10.3389/fphar.2025.1514997

**Published:** 2025-04-02

**Authors:** Ni Zhang, Xinchen Tian, Fen Liu, Xiaohan Jin, Jiaqi Zhang, Lingli Hao, Shulong Jiang, Qingbin Liu

**Affiliations:** ^1^ Cheeloo College of Medicine, Shandong University, Jinan, Shandong, China; ^2^ Clinical Medical Laboratory Center, Jining First People’s Hospital, Jining Medical University, Jining, Shandong, China; ^3^ Institute of Traditional Chinese Medicine, Shandong University of Traditional Chinese Medicine, Jinan, Shandong, China; ^4^ Jining No. 1 People’s Hospital, Shandong First Medical University, Jining, China; ^5^ Center for Post-Doctoral Studies, Shandong University of Traditional Chinese Medicine, Jinan, China

**Keywords:** network pharmacology, molecular docking, curcumol, hepatocellular carcinoma, sorafenib-resistance

## Abstract

**Background:**

Curcumol, a bioactive sesquiterpenoid extracted from traditional Chinese medicine (TCM), has demonstrated potential in overcoming tumor drug resistance. However, its mechanisms in reversing drug resistance, particularly in hepatocellular carcinoma (HCC) resistant to sorafenib, are not yet fully elucidated. This study aims to explore the molecular mechanisms by which curcumol reverses sorafenib resistance in HCC using a combination of network pharmacology, molecular docking, and *in vivo* and *in vitro* experiments.

**Methods:**

We identified curcumol targets and genes associated with sorafenib-resistant HCC, resulting in a set of overlapping targets. These intersection targets underwent enrichment analysis using DAVID, and a protein-protein interaction (PPI) network was constructed via the STRING database and Cytoscape. Molecular docking confirmed the binding of curcumol to core targets. *In vitro* assays, including CCK-8, colony formation assay, apoptosis detection, wound healing, and Transwell assays, evaluated curcumol’s effects on sorafenib-resistant HCC cells. Western blotting assessed the impact on PI3K/AKT and JAK/STAT3 signaling pathways. Additionally, a sorafenib-resistant HCC xenograft mouse model was established to observe the *in vivo* efficacy of curcumol combined with sorafenib.

**Results:**

We identified 117 potential targets for curcumol in reversing sorafenib resistance in HCC. Among them, five core targets—ALB, STAT3, HSP90AA1, HSP90AB1, and SRC—showed strong binding affinity with curcumol. KEGG pathway analysis of the intersecting genes highlighted significant involvement of the PI3K/AKT, JAK/STAT3, Ras, Rap1, HIF-1, FoxO, and mTOR signaling pathways. *In vitro* experiments revealed that curcumol significantly enhanced the sensitivity of sorafenib-resistant HCC cells to sorafenib, inhibiting cell proliferation, invasion, and migration while promoting apoptosis. *In vivo* studies further confirmed that curcumol combined with sorafenib effectively inhibited tumor growth in sorafenib-resistant HCC models.

**Conclusion:**

This study provides compelling evidence that curcumol can reverse sorafenib resistance in HCC by modulating multiple signaling pathways, including PI3K/AKT and JAK/STAT3 pathways. Our findings suggest that curcumol holds promise as a novel therapeutic agent for overcoming drug resistance in HCC, offering a new avenue for clinical intervention.

## Introduction

Hepatocellular carcinoma (HCC) is the most common primary liver malignancy and ranks as the fourth leading cause of cancer-related death worldwide (Author Anonymous, 2021; Bray et al., 2024). It usually appears in patients with chronic liver inflammation associated with viral infections, alcohol overuse, or metabolic syndrome (Bray et al., 2024). Unfortunately, more than 50% of HCC cases are diagnosed at an advanced stage, where treatment options, including molecular targeted cancer therapies, are limited (Marrero et al., 2018; Author Anonymous, 2021). Sorafenib, a multi-target tyrosine kinase inhibitor (TKI), was the first FDA-approved targeted therapy for advanced HCC (Llovet et al., 2008; Roskoski, 2023). Although studies have shown that sorafenib can extend the median overall survival of patients with advanced HCC by 2–3 months, its clinical benefits are significantly limited by the development of resistance within 6 months of treatment initiation (Tang et al., 2020).

Given these challenges, there is a pressing need to explore strategies to overcome sorafenib resistance and develop more effective combination therapies. Traditional Chinese Medicine (TCM), with a history spanning thousands of years, has been an essential part of cancer prevention and treatment in China (Xu et al., 2013; Nie et al., 2016). TCM remains a vital adjunctive therapy in cancer treatment due to its efficacy, minimal side effects, wide availability, and cost-effectiveness (Wang et al., 2020; Zhang et al., 2021; Li et al., 2025; Xi et al., 2025). Among the many compounds used in TCM, curcumol, a bioactive sesquiterpene derived from Zingiberaceae plants, has demonstrated potent antitumor, antibacterial, antioxidant, and anti-inflammatory effects across various cancers, without causing significant adverse effects (Wei et al., 2019). Notably, curcumol has shown potential in enhancing the efficacy of chemotherapy, particularly in cases of chemoresistance. For instance, curcumol has been reported to increase the sensitivity of triple-negative breast cancer cells to doxorubicin and enhance the effectiveness of cisplatin in gastric cancer cells (Huang et al., 2020; Zeng et al., 2020).

Despite its broad-spectrum pharmacological activities, the specific mechanisms by which curcumol reverses drug resistance in cancer, especially in sorafenib-resistant HCC, remain largely unexplored. Therefore, this study aims to elucidate the underlying mechanisms of curcumol’s effects on sorafenib-resistant HCC by leveraging network pharmacology and *in vivo* and *in vitro* experiments. Network pharmacology, an emerging field in TCM research, integrates bioinformatics, chemical informatics, and systems biology to unravel the complex interactions between drugs, targets, and diseases (Nogales et al., 2022). By applying this approach, we aim to provide a theoretical foundation for the combined use of curcumol and sorafenib in treating sorafenib-resistant HCC.

## Materials and methods

### Network pharmacology

#### Collection of sorafenib-resistant and curcumol-related targets

Table 1 listed the databases utilized in this study. The 2D chemical structure and SMILES format of curcumol were retrieved from the PubChem database (Kim et al., 2021). Curcumol targets were identified using the Swiss Target Prediction database (Daina et al., 2019). The GEO database’s GSE109211 dataset was selected, focusing on HCC patients who are sensitive or resistant to sorafenib (21 sorafenib treatment responders and 46 non-responders) (Clough and Barrett, 2016; Pinyol et al., 2019). Differentially expressed genes were identified using GEO2R with the criteria of adj.P.Val <0.05 and |log2 (fold change) (FC)| > 1. Sorafenib-resistant HCC-related genes were further screened from the GeneCards database using the keyword “sorafenib-resistant hepatocellular carcinoma” (Stelzer et al., 2016). The obtained genes related to sorafenib-resistant HCC were combined and duplicates were removed to obtain targets for sorafenib-resistant HCC.

**TABLE 1:** The basic information of the databases used for screening curcumol in reversing sorafenib resistance in HCC.

Name	URL
PubChem	https://pubchem.ncbi.nlm.nih.gov/
SwissTargetPrediction	http://www.swisstargetprediction.ch/
GEO	https://www.ncbi.nlm.nih.gov/geo/
GeneCards	https://www.genecards.org/
Venny 2.1.0	https://bioinfogp.cnb.csic.es/tools/venny/index.html
STRING	https://cn.string-db.org/
Cytoscape	https://cytoscape.org/
DAVID	https://david.ncifcrf.gov/
HPA	https://www.proteinatlas.org/
GEPIA	http://gepia.cancer-pku.cn/
Kaplan-Meier plotter	https://kmplot.com/
TIMER	https://cistrome.shinyapps.io/timer/

#### Screening of common drug-disease targets and construction of the PPI network

Venny2.0 was utilized to identify overlapping targets between curcumol and sorafenib-resistant HCC. These overlapping genes represent potential targets for curcumol in treating sorafenib-resistant HCC. The STRING database was employed to construct a protein-protein interaction (PPI) network for these common targets (Ding and Kihara, 2019; Szklarczyk et al., 2023). The tsv file was then imported into Cytoscape 3.9.0 for visualization (Shannon et al., 2003).

#### GO and KEGG analysis

GO and KEGG analyses were conducted on curcumol targets in sorafenib-resistant HCC using the “Functional Annotation” tool on the DAVID website (Huang et al., 2009). The data obtained were visualized using bioinformatics tools (https://www.bioinformatics.com.cn/).

#### Core target screening

In Cytoscape 3.9.0, the “cytoHubba” plugin was employed to filter the top 10 targets based on Degree, Betweenness, Maximum Neighborhood Component (MNC), Maximal Clique Centrality (MCC), and Closeness. The intersection of the targets obtained from these five calculation methods was considered as core targets.

#### Molecular docking

The SDF format file of the core drug’s active components was obtained from the PubChem database, and key target protein structures were collected from the PDB database. Pymol-2.1.0 software was used to optimize the targets by removing water molecules and small molecule ligands (Seeliger and de Groot, 2010). Hydrogenation and charge treatments were performed using AutoDock Tools-1.5.6, and the structures were saved in pdbqt format (Morris et al., 2009). Molecular docking was conducted using the key targets as receptors and their corresponding active components as ligands with the vina-2.0 tool in Pyrx software to calculate binding energy and output result files.

### Additional validation of core genes

#### Validation of core target protein expression levels

The Human Protein Atlas database was used to analyze the expression levels of core target proteins in HCC tissues and normal liver tissues (Uhlén et al., 2015).

#### Validation of core target gene expression levels

The mRNA expression levels and pathological stages of core targets were validated using the “Expression DIY” tool in GEPIA (Tang et al., 2017).

#### Validation of core target prognosis

To assess the impact of core target proteins on the prognosis of HCC patients, the correlation between their expression levels and overall survival was analyzed using the Kaplan-Meier plotter.

#### Immune cell infiltration levels of core targets

The TIMER database was used to explore the relationship between core targets and immune infiltration levels in HCC, thereby elucidating the potential mechanisms of the immune microenvironment (Li et al., 2017).

### Experimental validation

#### Cell culture and viability assay

Huh7-R and Hep-G2-R cells were cultured in DMEM (Gibco) containing 10% fetal bovine serum and 1% penicillin-streptomycin (Gibco) at 37°C with 5% CO_2_. Cells were seeded at a density of 8 × 10^3^ cells per well in 96-well plates for viability assays. After overnight incubation, cells were treated with various concentrations of sorafenib, curcumol, or their combination for 24 h. Subsequently, 100 μL of DMEM (without FBS) containing 10 μL CCK-8 solution was added to each well. Absorbance was measured at 460 nm after 1 h of incubation in the dark at 37°C.

#### Colony formation

For colony formation assays, cells in the logarithmic growth phase were seeded in 6-well plates (1,000 cells/well) and treated with curcumol, sorafenib, or their combination. A control group was also included. After 14 days, cells were fixed with 2 mL of 4% paraformaldehyde for 15 min and stained with 0.1% crystal violet for 15 min.

#### Apoptosis

Apoptosis was detected using Annexin V/7-AAD according to the manufacturer’s protocol (BioLegend). Cells were treated with curcumol, sorafenib, or their combination for 24 h, with a control group included, and then resuspended in Annexin V binding buffer. The cells were stained with 5 μL Annexin V and 5 μL 7-AAD and analyzed by flow cytometry.

#### Cell scratch, invasion, and migration assays

For the cell scratch assay, cells were seeded in 6-well plates for 48 h. A scratch was made in the cell monolayer using a pipette tip. Cells were treated with curcumol, sorafenib, or their combination, with a control group included. Photographs were taken at 0, 24, and 48 h after scratching. For the invasion assay, a Matrigel-coated Transwell chamber (Corning, United States) was used to detect the number of invading cells as per the manufacturer’s instructions. Cells were treated with curcumol, sorafenib, or their combination, with a control group included. After 48 h of culture, cells were fixed with 2 mL anhydrous formaldehyde for 15 min and stained with 0.1% crystal violet for 15 min. For the migration assay, the number of migrating cells was measured using a Transwell chamber (Corning, United States). Cells were treated with curcumol, sorafenib, or their combination, with a control group included. After 48 h of culture, cells were fixed with 2 mL anhydrous formaldehyde for 15 min and stained with 0.1% crystal violet for 15 min.

#### Western blotting

Cells were seeded in 6-well plates, and after various treatments, tumor cells were lysed with RIPA buffer, and proteins were extracted. Protein concentration was determined using the BCA assay (Beyotime). SDS-PAGE was used to separate proteins, which were then transferred to PVDF membranes and incubated with primary antibodies. The following primary antibodies were used: PI3K (1:1,000, CST), p-PI3K (1:1,000, Abcam), AKT (1:1,000, CST), p-AKT (Ser473) (1:2,000, CST), JAK2 (1:1,000, CST), p-JAK2 (1:1,000, CST), STAT3 (1:2,000, Proteintech), p-STAT3 (1:2,000, BOSTER), β-actin (1:10,000, Affinity). After overnight incubation at 4°C, secondary antibodies (1:1,000) were incubated at room temperature for 1 h. Membranes were observed using an enhanced chemiluminescence Western blot detection system.

#### Mouse tumor xenograft assay

All animal experiments were approved by the Animal Ethics Committee of the First People’s Hospital of Jining. Curcumol was purchased from Shanghai Topscience Co., Ltd. (Shanghai, China). Nude mice (4 weeks old, male, 20.0 ± 5.0 g) were purchased from Jinan Pengyue Experimental Animal Breeding Co., Ltd. (Shandong, China). The animals were housed under standard SPF conditions. Sorafenib-resistant Huh7-R cell suspension (0.2 mL per mouse, 2 × 10^7^ cells/mL) was injected subcutaneously into the right axilla of the nude mice. Tumor volume was calculated as (length × width^2^) × 0.5. When the average tumor volume increased to 100 mm^3^, the mice were randomly divided into four groups: model group, curcumol group (40 mg/kg) (Yang et al., 2022), sorafenib group (30 mg/kg), and curcumol combined with sorafenib group, with 5 mice in each group. Curcumol was administered intraperitoneally every other day, and sorafenib was administered by oral gavage once daily. Mice in the model group received an injection of 0.2 mL physiological saline. After 2 weeks of continuous intervention, the nude mice were weighed, sacrificed, their xenografts were excised, weighed, photographed, and stored for further experiments.

#### Immunohistochemistry

Immunohistochemical staining was performed using Ki-67, p-AKT, and p-STAT3 antibodies according to the manufacturer’s instructions. TUNEL staining was conducted to analyze apoptosis in sorafenib-resistant HCC cells. The stained sections were observed under a light microscope, and images were captured for analysis.

#### Statistical analysis

The data were analyzed by GraphPad Prism 9. The results were presented as mean ± SEM, and statistical analysis was carried out using a one-way ANOVA test for multiple groups. A p-value <0.05 was considered statistically significant.

## Results

### Network pharmacology

#### Curcumol-related targets and sorafenib-resistant hepatocellular carcinoma-related targets

**FIGURE 1:**
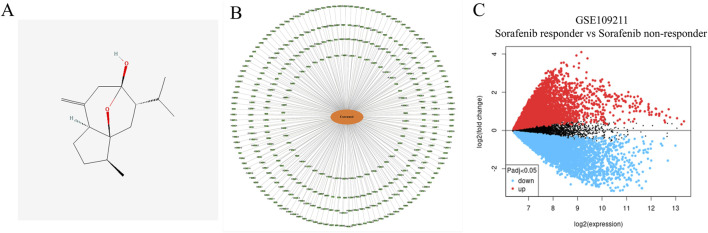
Targets relevant to the treatment of Sorafenib-resistant HCC and Curcumol. **(A)** The molecular structures of Curcumol. **(B)** Targets of Curcumol. **(C)** Volcano plot of DEGs associated with Sorafenib-resistant HCC.

The chemical structure of curcumol, which plays a critical role in traditional Chinese medicine, is depicted in Figure 1A. We identified 348 potential targets related to curcumol. These targets were then mapped to create a “Curcumol Targets” network, visualized using Cytoscape software (Figure 1B).

The GSE109211 dataset was selected from the GEO database to identify differentially expressed genes in HCC patients who are either sensitive or resistant to sorafenib. Genes meeting the criteria of |log FC| > 1 and adjusted p-value <0.05 were classified as differentially expressed and were visualized using a volcano plot (Figure 1C). Sorafenib-resistant HCC-related targets were further obtained from the GeneCards database. By intersecting the data from the GSE109211 dataset with the targets from the GeneCards database and removing duplicates, we identified the specific targets associated with sorafenib-resistant HCC.

#### Target analysis and PPI network of curcumol in the treatment of sorafenib-resistant hepatocellular carcinoma

**FIGURE 2:**
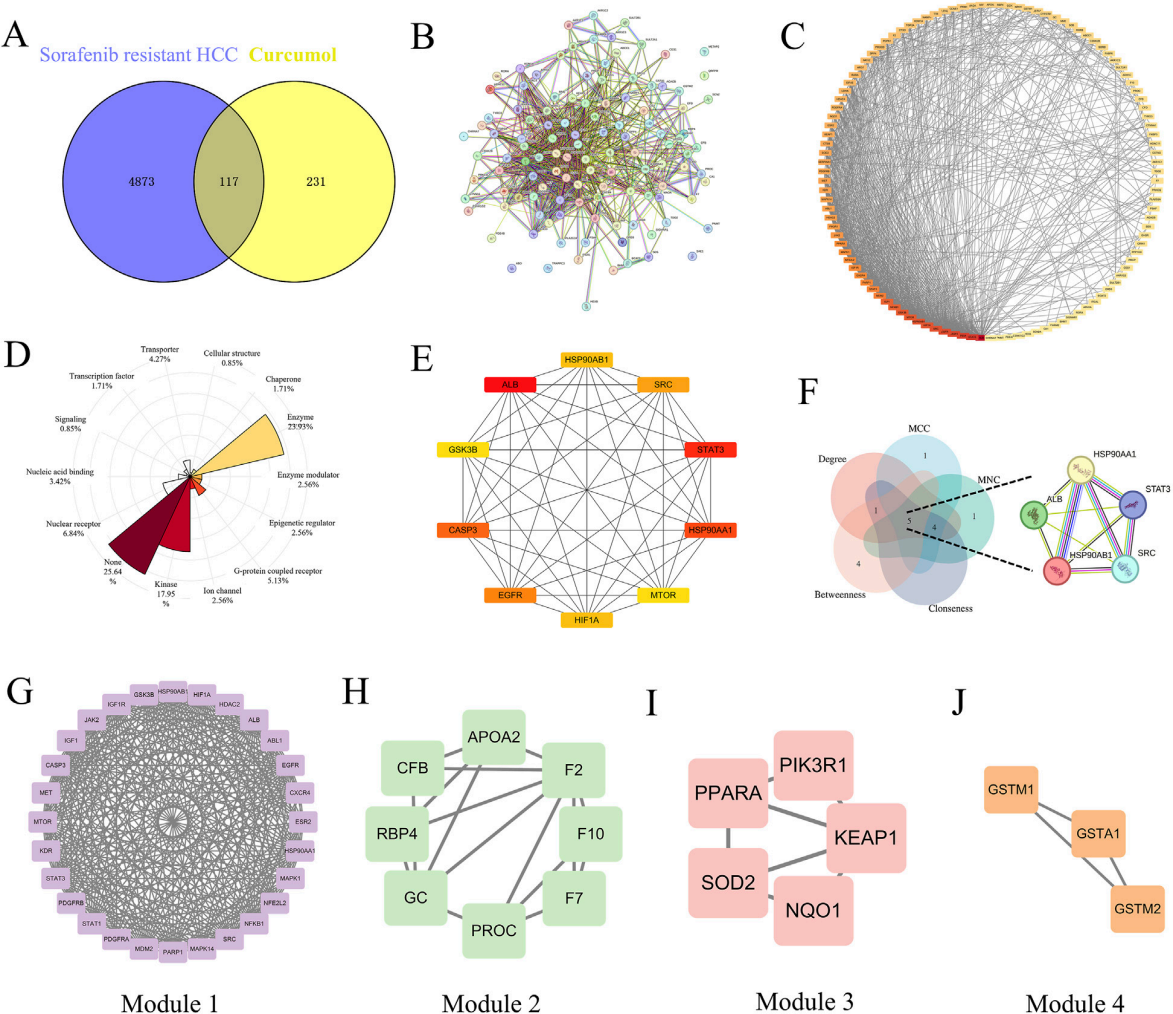
The therapeutic targets and PPI network analysis. **(A)** Venn diagram of common genes between Sorafenib-resistant HCC and Curcumol. **(B)** PPI network map of 117 target genes. **(C)** PPI network analysis based on Degree ranking. **(D)** Rose plot for showing the proportion of gene attributes of therapeutic targets. **(E)** Top 10 vital target genes network based on Degree ranking. **(F)** Venn diagram of core targets screened by five topological parameters. **(G–J)** Four significant modules generated by the MCODE plugin.

To identify common targets between curcumol and sorafenib-resistant HCC, we used Venny 2.1.0 software to match 4,990 disease-related targets with 348 drug-related targets, resulting in 117 overlapping targets for further investigation (Figure 2A). These common targets represent the potential therapeutic targets of curcumol in addressing sorafenib-resistant HCC. A protein-protein interaction (PPI) network for these common targets was constructed using the STRING database (Figure 2B) and visualized in Cytoscape (Figure 2C). DisGeNet analysis revealed that the therapeutic targets exhibited multifaceted gene properties, suggesting that curcumol exerts multi-layered regulatory effects on sorafenib-resistant HCC cells (Figure 2D). Kinases and enzymes constituted a significant proportion of these targets.

In our PPI network analysis, the top 10 therapeutic targets were filtered based on their Degree values, including ALB (Degree = 64), STAT3 (Degree = 50), HSP90AA1 (Degree = 49), CASP3 (Degree = 47), EGFR (Degree = 46), SRC (Degree = 45), HSP90AB1 (Degree = 44), HIF1A (Degree = 44), mTOR (Degree = 43), and GSK3B (Degree = 43) (Figure 2E). The intersection of the top 5 targets filtered by Degree, Maximum Neighborhood Component (MNC), Maximal Clique Centrality (MCC), Closeness, and Betweenness was identified as core targets, including ALB, STAT3, HSP90AA1, SRC, and HSP90AB1 (Figure 2F).

Using the MCODE plugin, four clustering modules of the PPI network were established (Modules 1, 2, 3, and 4, with scores of 23.852, 4.571, 3.5, and 3, respectively), each with distinct roles (Figures 2G–J). The clustering score represents the core density of nodes and topologically adjacent nodes; higher scores indicate more concentrated clusters. Module 1, which contained the most target genes and had the highest score, was identified as the most significant functional module.

#### GO and KEGG enrichment analysis

**FIGURE 3:**
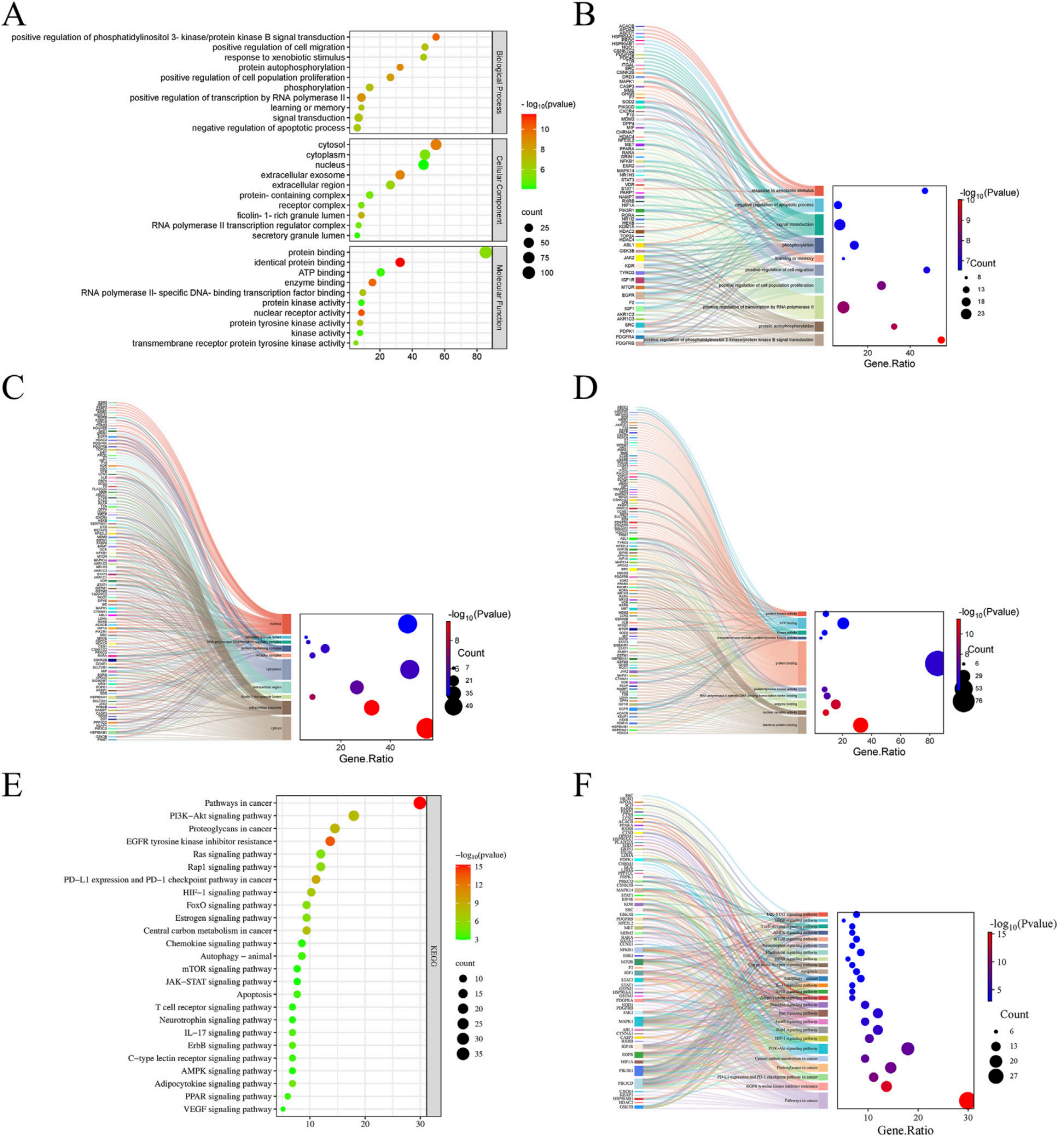
GO and KEGG pathway enrichment analyses. **(A)** GO functional enrichment analysis of Curcumol in Sorafenib-resistant HCC. **(B)** The Sankey plot showing GO BP. **(C)** The Sankey plot showing GO CC. **(D)** The Sankey plot showing GO MF. **(E)** KEGG enrichment analysis of potential signaling pathways associated with Curcumol in Sorafenib-resistant HCC. **(F)** The Sankey plot showing KEGG pathways.

To systematically explore the complex mechanisms by which curcumol affects sorafenib-resistant HCC, GO functional annotation and KEGG pathway enrichment analyses were conducted on the 117 intersecting genes. A total of 500 entries were obtained, including 340 in Biological Process (BP), 58 in Cellular Component (CC), and 102 in Molecular Function (MF) categories. Bubble plots were generated for the top 10 enriched terms in BP, CC, and MF categories (Figures 3A–D). The BP enrichment terms included “positive regulation of phosphatidylinositol 3-kinase/protein kinase B signal transduction”, “positive regulation of cell migration”, and “response to xenobiotic stimulus” (Figure 3B). The CC enrichment terms included “cytosol”, “cytoplasm”, and “nucleus” (Figure 3C). The MF enrichment terms included “protein binding”, “identical protein binding”, and “ATP binding” (Figure 3D).

KEGG pathway enrichment analysis identified 136 enriched signaling pathways, with the top 25 pathways involved in cancer-related processes displayed as a bubble plot (Figure 3E). The results demonstrated that curcumol is involved in several key signaling pathways related to sorafenib-resistant HCC, including “Pathways in cancer”, “PI3K-Akt signaling pathway”, “HIF-1 signaling pathway”, “Ras signaling pathway”, “mTOR signaling pathway”, and “JAK-STAT signaling pathway” (Figures 3E, F).

#### Molecular docking validation

**FIGURE 4:**
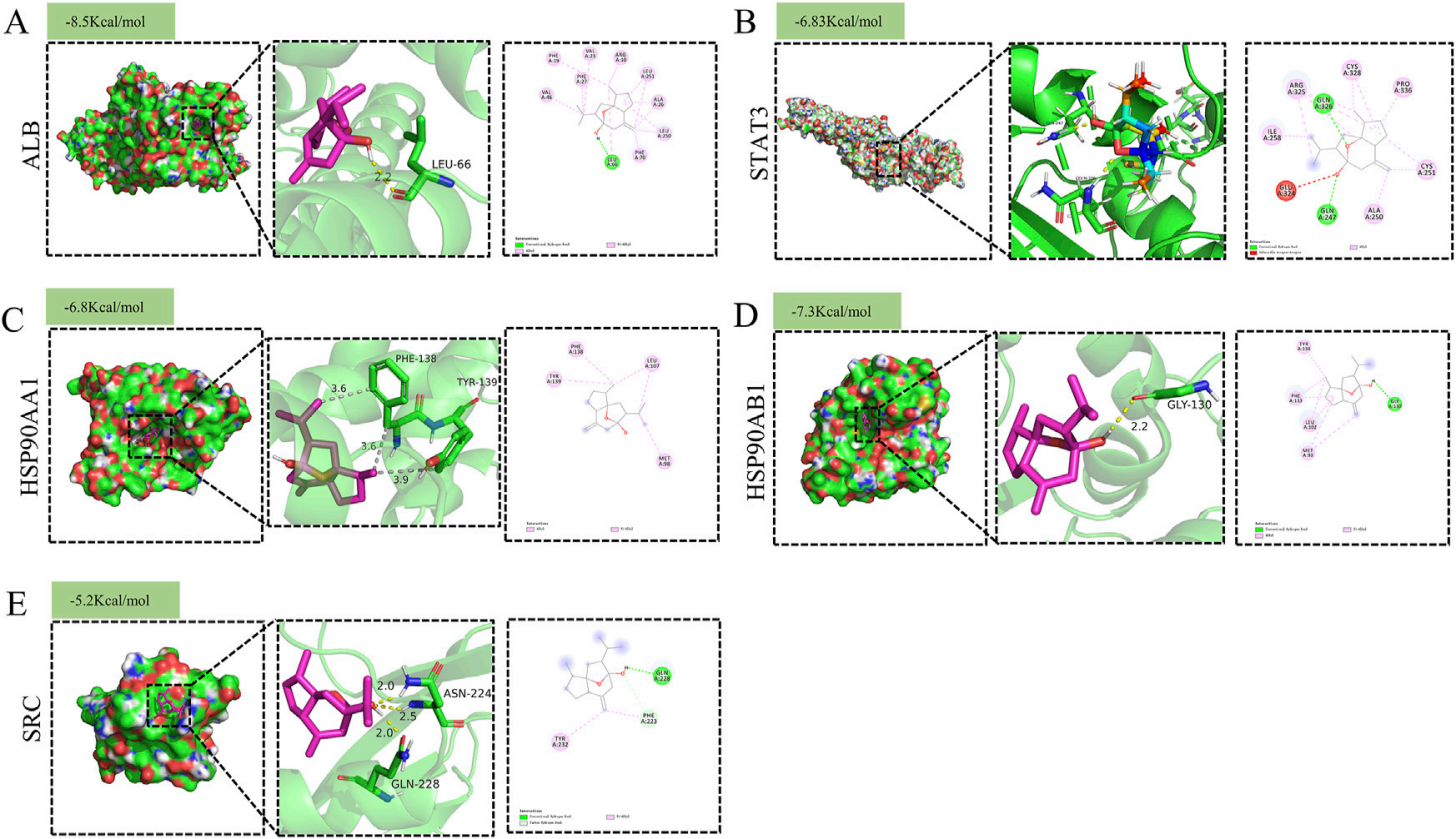
Molecular docking pattern of Curcumol with target proteins. **(A)** ALB **(B)** STAT3 **(C)** HSP90AA1 **(D)** HSP90AB1 **(E)** SRC.

To further validate the reliability of the network pharmacology predictions, molecular docking analysis was performed between curcumol and the identified core targets. Figures 4A–E illustrates the molecular docking between curcumol and the five core target proteins, along with their respective binding energies. The binding affinity between the ligand and the receptor is generally determined by the binding energy, with lower energy levels indicating stronger binding. Typically, a binding energy score (kcal/mol) of less than −5 is considered indicative of strong binding affinity (Wang et al., 2024). Our analysis showed that the binding energies between curcumol and the core genes were all ≤ −5 kcal/mol, suggesting that curcumol can easily bind to these targets and maintain relatively stable conformations (Figures 4A–E). These findings suggest that the five core targets play a critical role in tumor progression and may be key to curcumol’s mechanism of reversing sorafenib resistance in HCC.

### Clinical significance of core target genes

#### Protein expression levels of core genes

**FIGURE 5:**
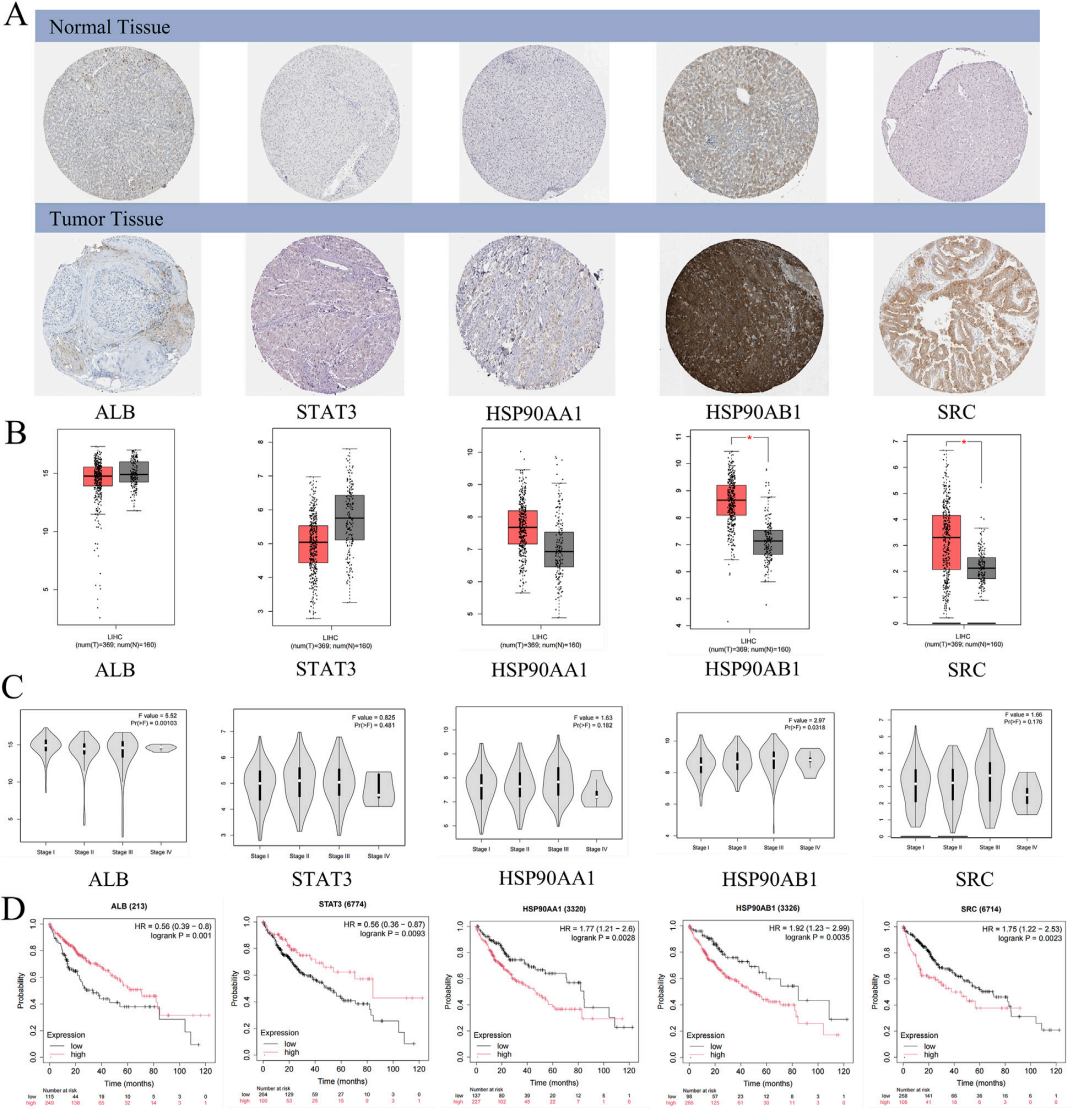
Clinical significance of core targets. **(A)** The expression of core targets at the protein levels in normal and HCC samples. **(B)** The expression of core targets at the transcriptome levels in normal and HCC samples. T: HCC tumor tissue, N: normal tissue. **(C)** The expression level of core targets at different stages of HCC. **(D)** The correlation between core target expression level and HCC prognosis.

Immunohistochemical analysis retrieved from the Human Protein Atlas (HPA) database revealed that the expression levels of HSP90AB1 and SRC proteins were significantly elevated in HCC tissues compared to normal liver tissues (Figure 5A). However, no significant differences were observed in the expression levels of ALB, STAT3, and HSP90AA1 between HCC and normal liver tissues (Figure 5A). This suggests that HSP90AB1 and SRC may play a critical role in the progression of HCC, potentially serving as biomarkers or therapeutic targets.

#### mRNA levels of core genes

mRNA expression levels of the core genes were validated using the GEPIA database, which showed that HSP90AB1 and SRC mRNA levels were significantly higher in HCC tissues compared to normal liver tissues (Figure 5B). Although STAT3, ALB, and HSP90AA1 exhibited differential expression between HCC tissues and normal tissues, these differences were not statistically significant (p > 0.01). Interestingly, ALB and HSP90AB1 expression varied significantly across different pathological stages of HCC, indicating its potential role in disease progression (Figure 5C). Conversely, STAT3, HSP90AA1, and SRC did not show significant stage-dependent differences (p > 0.01), suggesting a more consistent role across various stages of HCC (Figure 5C).

#### Relationship between core proteins and HCC survival rates

Kaplan-Meier survival analysis revealed that high expression levels of HSP90AA1, HSP90AB1, and SRC were associated with poor prognosis in HCC patients (p < 0.05), highlighting their potential as prognostic biomarkers (Figure 5D). In contrast, low expression levels of ALB and STAT3 were linked to unfavorable outcomes (p < 0.05), emphasizing the complex role of these proteins in HCC pathogenesis and their potential utility in patient-personalized treatment strategies (Figure 5D).

#### Relationship between core proteins and immune cell infiltration

**FIGURE 6:**
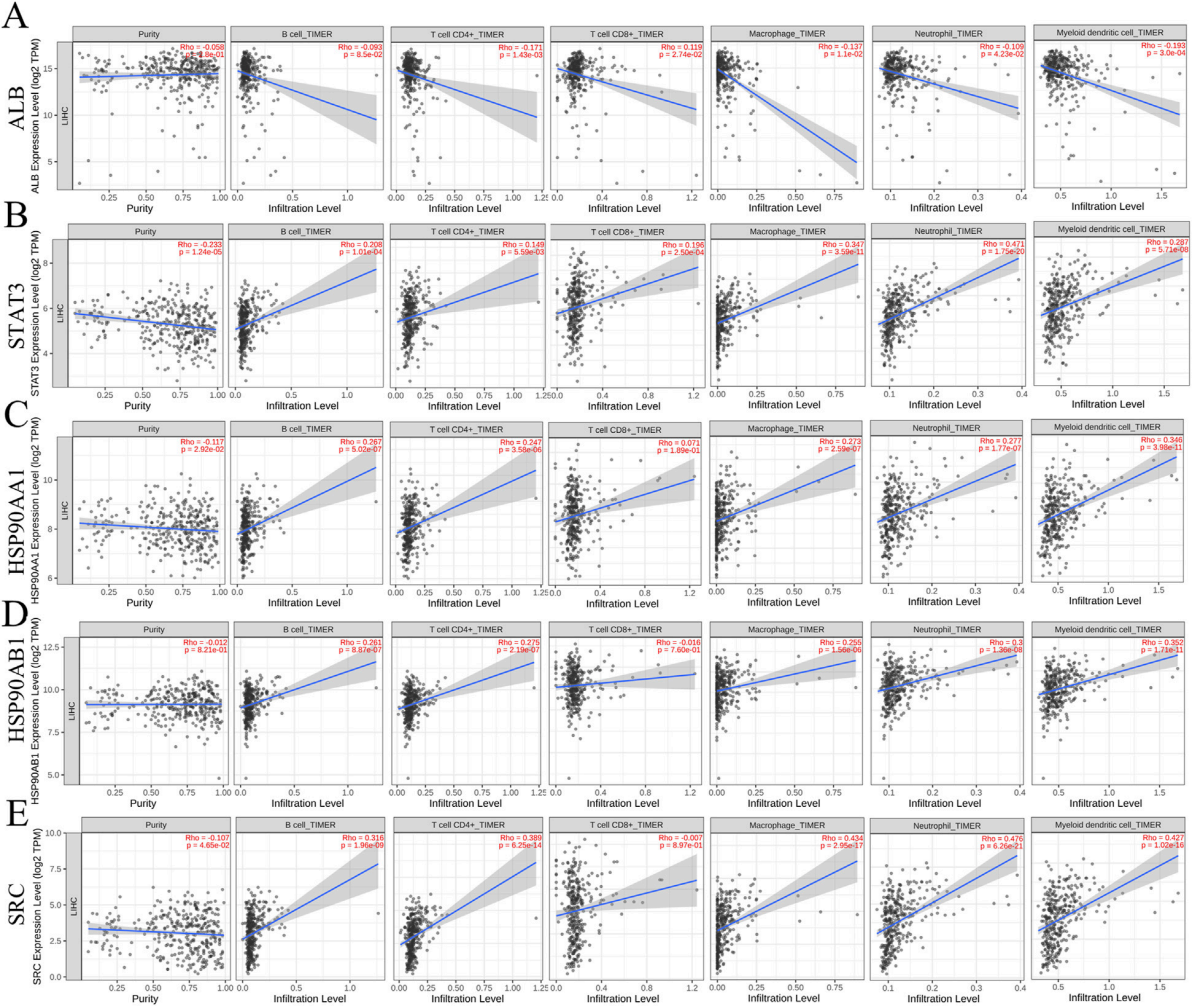
Relationship between differentially expressed core targets and immune cell infiltration. **(A)** ALB **(B)** STAT3 **(C)** HSP90AA1 **(D)** HSP90AB1 **(E)** SRC.

Correlation analysis between the core genes and immune cell infiltration, as analyzed via the TIMER database, demonstrated that all five core molecules were negatively correlated with tumor purity (Figures 6A–E). Specifically, ALB levels were inversely correlated with the infiltration of B cells, CD4^+^ T cells, macrophages, neutrophils, and dendritic cells, but positively correlated with CD8^+^ T cell infiltration (Figure 6A). Additionally, STAT3 and HSP90AA1 levels were positively correlated with the infiltration of B cells, CD4^+^ T cells, CD8^+^ T cells, macrophages, neutrophils, and dendritic cells (Figures 6B, C). HSP90AB1 and SRC showed similar trends, with positive correlations with the infiltration of B cells, CD4^+^ T cells, macrophages, neutrophils, and dendritic cells, but negative correlations with CD8^+^ T cell infiltration (Figures 6D, E). Our next analysis focuses on these five core genes. The Cox proportional risk model was used to analyze the clinical significance of core genes and immune infiltrating cells. Our analysis showed that stage 3, stage 4, macrophages, neutrophils, HSP90AA1, HSP90AB1, and SRC were significantly associated with overall survival in LIHC patients (Table 2). These findings suggest that the core targets may influence the immune microenvironment of HCC, potentially impacting tumor progression and response to therapy.

**TABLE 2:** Analysis of tumor infiltrating immune cells and hub genes using the Cox proportional hazards model.

	Coef	HR	95%CI-I	95%CI-u	*p*.value	Significance
Age	0.011	1.011	0.995	1.027	0.184	
Stage2	0.321	1.379	0.827	2.301	0.219	
Stage3	0.955	2.599	1.641	4.116	0	***
Stage4	1.561	4.763	1.412	16.073	0.012	*
Gendermale	−0.17	0.844	0.537	1.326	0.461	
RaceBlack	0.941	2.561	0.969	6.77	0.058	
RaceWhite	−0.012	0.988	0.619	1.576	0.959	
Purity	0.484	1.622	0.632	4.166	0.315	
B cell	−0.778	0.459	0.03	7.029	0.576	
CD4 T cell	0.246	1.279	0.109	14.982	0.845	
CD8 T cell	0.397	1.488	0.245	9.035	0.666	
Macrophage	3.701	40.478	5.831	280.986	0	***
Neutrophil	4.754	116.014	1.686	7982.097	0.028	*
Myeloid dendritic cell	1.067	2.906	1.037	8.139		
ALB	−0.063	0.939	0.856	1.03	0.183	
STAT3	0.073	1.075	0.863	1.34	0.518	
HSP90AA1	0.449	1.567	1.236	1.987	0	***
HSP90AB1	0.346	1.413	1.118	1.785	0.004	**
SRC	0.219	1.245	1.079	1.437	0.003	**

### Experimental validation

#### Combined treatment with curcumol and sorafenib inhibits cell proliferation and promotes apoptosis

**FIGURE 7:**
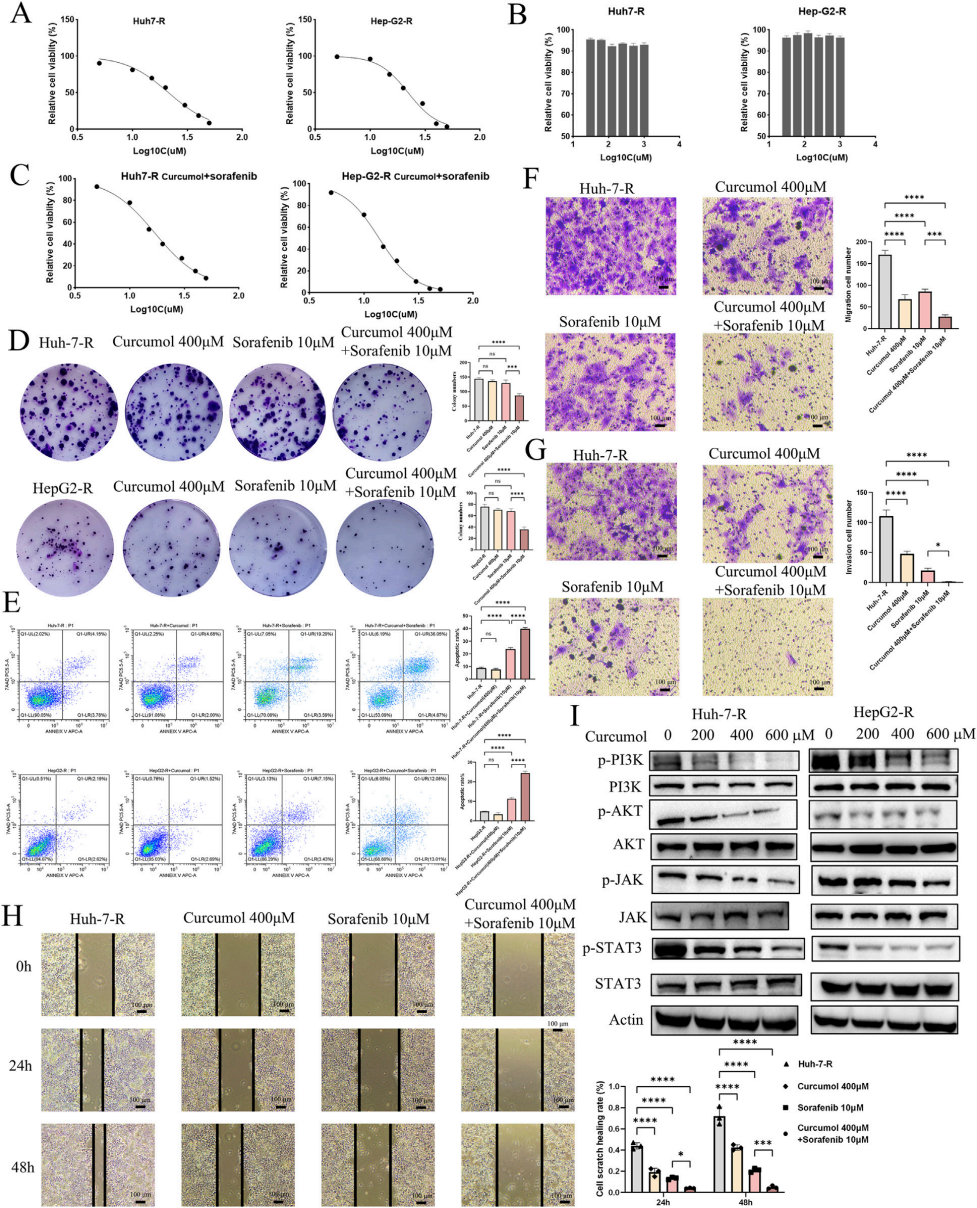
Experimental verification of the function of Curcumol in Sorafenib-resistant HCC cells. **(A)** Effect of Sorafenib (5, 10, 15, 20, 30, 40, and 50 μM) on Huh7-R and HepG2-R cells viability. **(B)** Effect of Curcumol (31.25, 62.5, 125, 250, 500, and 1000 μM) on Huh7-R and HepG2-R cells viability. **(C)** Effect of Curcumol combined with Sorafenib (5, 10, 15, 20, 30, 40, and 50 μM) on Huh7-R and HepG2-R cells viability. **(D)** Colony formation assay of Huh7-R and HepG2-R cells treated with Curcumol, Sorafenib, and Curcumol combined with Sorafenib, respectively. **(E)** Flow cytometry detection of apoptosis analysis of Huh7-R and HepG2-R cells treated with Curcumol, Sorafenib, and Curcumol combined with Sorafenib, respectively. **(F)** Transwell assay was performed to detect the effect of Curcumol on the migratory ability of Huh7-R cells. **(G)** Transwell assay to detect the effect of Curcumol on the invasive ability of Huh7-R cells. **(H)** Cell scratch assay of Huh7-R cells treated with Curcumol, Sorafenib, and Curcumol combined with Sorafenib, respectively. **(I)** The protein expression levels of PI3K\AKT pathway and JAK/STAT3 pathway following intervention with Curcumol combined with Sorafenib. *p < 0.05, **p < 0.01, ***p < 0.001.

Following the network pharmacology analysis, the inhibitory effects of curcumol on sorafenib-resistant HCC cells were validated through the CCK-8 assay. The CCK8 assay demonstrated that sorafenib exhibited a dose-dependent inhibitory effect on sorafenib-resistant liver cancer cells, with IC50 values of 21.28 μM and 22.14 μM, respectively (Figure 7A). Further CCK8 assays revealed that curcumol alone did not significantly inhibit the proliferation of sorafenib-resistant liver cancer cells (Figure 7B). However, when co-administered with curcumol for 24 h, along with varying concentrations of sorafenib (5, 10, 15, 20, 30, 40, and 50 μM), the combined treatment enhanced the inhibitory effect of sorafenib (Figure 7C). The IC50 values of the combined treatment were reduced to 17.00 μM and 13.76 μM, indicating that curcumol potentiates the efficacy of sorafenib in overcoming resistance in liver cancer cells (Figure 7C). This was further corroborated by the colony formation assay, which showed a significant decrease in the proliferation of Huh7-R and Hep-G2-R cells under the combined treatment (Figure 7D). Additionally, flow cytometry analysis revealed that the combination of curcumol and sorafenib significantly increased the percentage of apoptotic cells compared to the control and single-agent treatments (Figure 7E). These results suggest that curcumol, when combined with sorafenib, effectively inhibits cell proliferation and promotes apoptosis in sorafenib-resistant HCC cells.

#### Curcumol and sorafenib synergistically suppress cell invasion and migration

The Transwell migration and invasion assays were performed to investigate the effects of curcumol and sorafenib on the migratory and invasive capabilities of Huh7-R cells. Curcumol and sorafenib co-treatment markedly reduced the migratory (Figure 7F) and invasive (Figure 7G) potential of sorafenib-resistant HCC cells. The scratch assay further supported these findings, demonstrating a significant reduction in wound closure in the combination treatment group (Figure 7H).

#### Western blot analysis confirms pathway inhibition

Given the significant enrichment of the PI3K/AKT pathway in the network pharmacology analysis, we conducted Western blotting to assess the effects of the combination treatment on key signaling pathways. The results showed a marked reduction in the phosphorylation levels of AKT (at Ser-473) and PI3K in the combination treatment group, suggesting that curcumol may reverse sorafenib resistance by targeting the PI3K/AKT pathway (Figure 7I). Additionally, the JAK/STAT3 signaling pathway was also downregulated in the combination treatment, as evidenced by decreased phosphorylation levels of JAK2 and STAT3 (Figure 7I). These findings imply that the anti-tumor effects of curcumol and sorafenib may be mediated through multiple signaling pathways, including PI3K/AKT and JAK/STAT3.

#### Curcumol and sorafenib combination reduces tumor growth in a xenograft model

**FIGURE 8:**
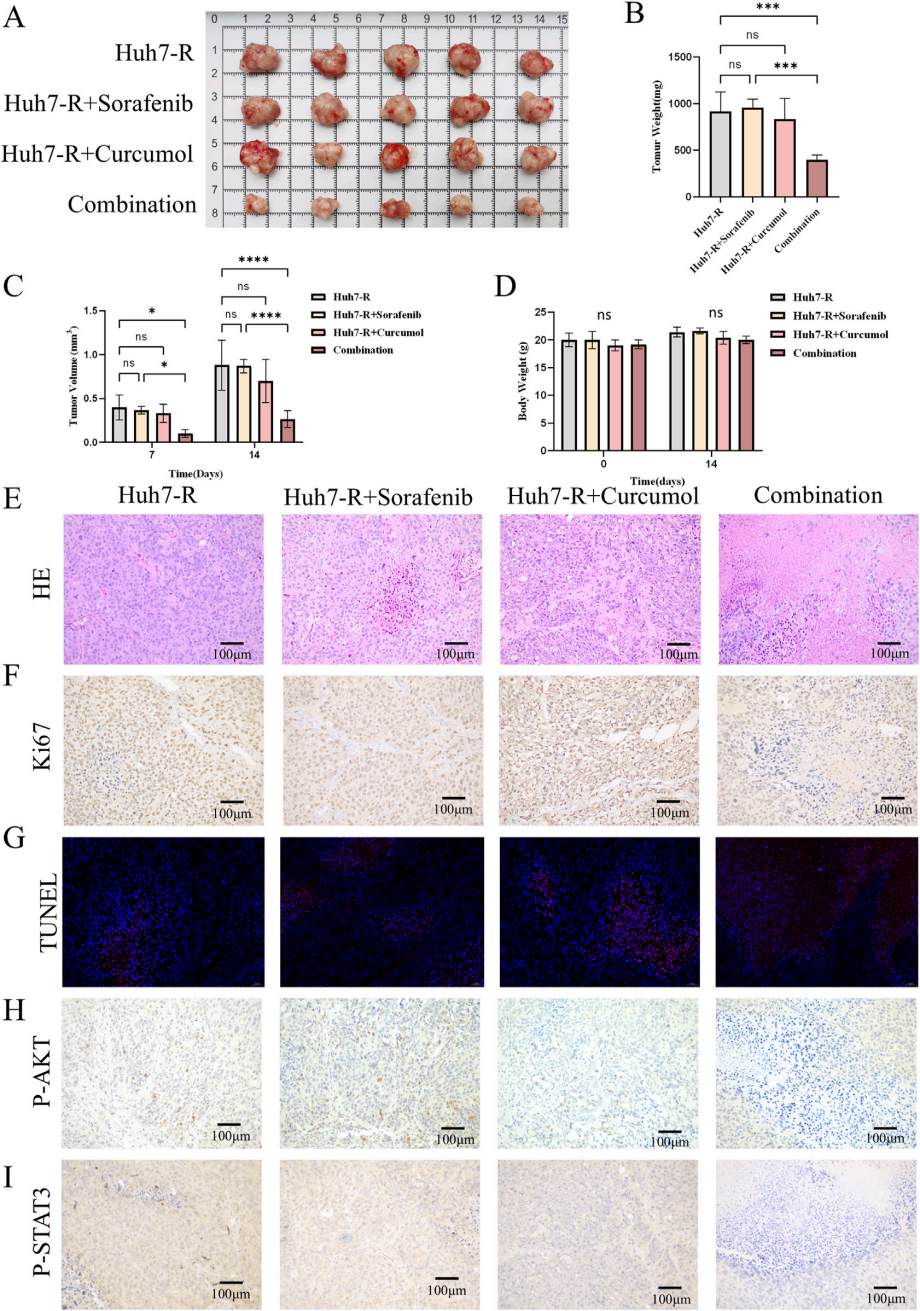
Curcumol combined with sorafenib inhibited the tumor growth of Huh7-R cell xenografts *in vivo*. **(A)** The gross appearance of the tumors in xenografted mice. **(B)** The tumor weight of xenografted mice after 14 days of intervention. **(C)** Tumor volumes in xenograft mice after 7 and 14 days of intervention. **(D)** Body weight of xenografted mice before and after 14 days of intervention. **(E)** HE staining of tumors. Scar bar = 100 μm. **(F)** Ki67 staining of tumors. Scar bar = 100 μm. **(G)** TUNEL staining of tumors. Scar bar = 50 μm. **(H, I)** Tumor tissues were stained for p-AKT **(H)**, and p-STAT3 **(I)**. Scar bar = 100 μm *p < 0.05, **p < 0.01, ***p < 0.001.

To validate these findings *in vivo*, the sorafenib-resistant HCC xenograft model was established in nude mice. The mice were divided into four groups: control, curcumol-treatment, sorafenib-treatment, and combination treatment. After 14 days of treatment, the combination group exhibited significantly reduced tumor size and weight compared to the other groups (Figures 8A–C). There was no significant change in the weight of the nude mice as shown in Figure 8D.

In the control, sorafenib-treatment, and curcumol-treatment groups, HE staining of the tumor sections showed dense, disorganized cellular structures with limited necrosis. In contrast, the combination group exhibited significant tumor necrosis and reduced tumor cell density, reflecting the enhanced antitumor effect of the combined treatment (Figure 8E). Histological analysis of the tumors revealed decreased cell proliferation and increased apoptosis in the combination treatment group, as shown by Ki67 and TUNEL staining (Figures 8F, G). Additionally, immunohistochemistry confirmed the downregulation of phosphorylated AKT and STAT3 in the tumors of the combination group (Figures 8H, I). These results provide further evidence that curcumol enhances the efficacy of sorafenib in overcoming resistance in HCC.

## Discussion

Hepatocellular carcinoma (HCC) is a highly lethal malignancy with increasing incidence worldwide. Although sorafenib, a multi-target tyrosine kinase inhibitor (TKI), has marked a significant advance in the treatment of advanced HCC (Llovet et al., 2008), its long-term efficacy is severely hampered by the rapid development of drug resistance (Tang et al., 2020). The mechanisms underlying sorafenib resistance are multifaceted, involving the dysregulation of several critical signaling pathways, notably the PI3K/AKT and JAK/STAT pathways, which are crucial for tumor cell proliferation, apoptosis, and metastasis (Zhu et al., 2017; Zhang et al., 2018). Addressing sorafenib resistance has therefore become a pivotal focus in current HCC research.

Curcumol, a sesquiterpene compound derived from plants of the family Zingiberaceae (Wei et al., 2019), has attracted considerable attention due to its potential role in reversing drug resistance in cancer therapy (Huang et al., 2020; Zeng et al., 2020; Gao et al., 2021; Liu, 2022). Known for its antitumor, antibacterial, antioxidant, and anti-inflammatory properties, curcumol has been shown to enhance the efficacy of chemotherapeutic agents like cisplatin and doxorubicin in various cancers, including breast and gastric cancer (Huang et al., 2020; Zeng et al., 2020). However, the specific mechanisms by which curcumol reverses sorafenib resistance in HCC have not been fully elucidated, warranting further investigation.

In this study, we employed a combination of network pharmacology, *in vivo*, and *in vitro* experiments to systematically explore the mechanisms by which curcumol reverses sorafenib resistance in HCC. Network pharmacology, an integrated approach that combines bioinformatics, chemoinformatics, and systems biology, allowed us to uncover the multi-target effects of curcumol and its interactions with sorafenib-resistant HCC cells.

Through the intersection analysis of 4,990 sorafenib-resistant genes and 348 curcumol-related genes, we identified 117 common targets. GO and KEGG enrichment analyses revealed that these targets are involved in several critical pathways, including the PI3K/AKT, HIF-1, Ras, mTOR, and JAK/STAT signaling pathways. These findings suggest that curcumol exerts a multidimensional regulatory effect on sorafenib-resistant HCC cells, providing a clear direction for subsequent experimental validation.

Our PPI network analysis identified five core genes—ALB, STAT3, HSP90AA1, HSP90AB1, and SRC—as potential key targets of curcumol in reversing sorafenib resistance. These genes are closely linked to vital cellular processes such as proliferation, differentiation, survival, adhesion, apoptosis, and signal transduction, making them promising therapeutic targets (Gj et al., 2005; Yu et al., 2014; Zuehlke et al., 2015; Haase and Fitze, 2016; Caner et al., 2021). Further molecular docking experiments confirmed strong binding affinities between curcumol and these core proteins, particularly within the PI3K/AKT pathway. Surface plasmon resonance (SPR) or isothermal titration calorimetry (ITC) validation of binding affinity will be pursued in future studies. These findings underscore the authenticity and predictive accuracy of our network pharmacology analysis.

*In vitro* experiments, including colony formation, apoptosis, CCK8, and Transwell assays, demonstrated that the combination of curcumol and sorafenib significantly inhibits the proliferation and invasion of sorafenib-resistant HCC cells while promoting apoptosis. Western blot analysis further revealed that this combination treatment downregulates the phosphorylation of key signaling molecules within the PI3K/AKT and JAK/STAT pathways, consistent with the predictions of our network pharmacology analysis.

The *in vivo* nude mouse xenograft model provided further validation of curcumol’s potential in reversing sorafenib resistance. Tumor size, weight, and volume were significantly reduced in the combination treatment group, with marked downregulation of phosphorylated AKT and STAT3 levels. These results provide strong experimental evidence supporting the clinical potential of curcumol as an adjunctive therapy to overcome sorafenib resistance in HCC.

Our study also has several limitations that should be acknowledged. First, while we focused on the PI3K/AKT and JAK/STAT3 pathways, the roles of other KEGG-identified pathways (e.g., Ras, mTOR) and their interactions remain unexplored. Future studies will use multi-omics approaches to systematically investigate these pathways and their contributions to curcumol’s effects. Second, the absence of patient-derived xenografts (PDXs) and clinical validation limits the translational relevance of our findings. We will incorporate PDX models and clinical samples in future work to better model sorafenib resistance. Third, although molecular docking and functional assays implicate core targets (e.g., STAT3, HSP90AB1), siRNA or CRISPR-based knockdown experiments are needed to establish causality. These validations will be prioritized to further clarify curcumol’s mechanism of action.

In comparison to other natural compounds explored for overcoming sorafenib resistance, curcumol demonstrates multi-target properties. For instance, piperlongumine, a natural alkaloid, has been shown to enhance sorafenib’s antitumor activity by mediating ROS-AMPK activation and targeting CPSF7 in liver cancer (Zheng et al., 2022). While piperlongumine primarily focuses on ROS-mediated pathways, curcumol exerts a broader regulatory effect by simultaneously modulating the PI3K/AKT, JAK/STAT, Ras and mTOR pathways, as demonstrated in our study. This multi-target action may provide a more comprehensive approach to reversing sorafenib resistance. Despite these promising findings, the clinical application of curcumol faces several challenges. The pharmacokinetic properties of curcumol, including bioavailability and plasma half-life, require optimization to ensure sufficient efficacy *in vivo*. Moreover, comprehensive safety and toxicity evaluations are crucial, particularly concerning high-dose or long-term use. Given the complexity of curcumol’s mechanisms, its efficacy may vary among patients, necessitating large-scale clinical trials to validate its generalizability across diverse populations.

In conclusion, this study systematically demonstrates, for the first time, that curcumol can reverse sorafenib resistance in HCC by modulating the PI3K/AKT and JAK/STAT signaling pathways. These findings not only provide new scientific evidence for curcumol as an adjunctive anticancer therapy but also offer novel insights into overcoming drug resistance in HCC. Future research should explore the combination of curcumol with other anticancer agents and its potential in reversing resistance in other cancer types. A deeper understanding of curcumol’s regulatory mechanisms will further support its development as a viable therapeutic option for drug-resistant HCC.

## Data Availability

Publicly available datasets were analyzed in this study. This data can be found here: GEO repository, accession number GSE109211.
